# Postpartum depression in outpatient care in Latvia: symptom prevalence and associated clinical and sociodemographic factors

**DOI:** 10.1192/j.eurpsy.2026.10600

**Published:** 2026-07-31

**Authors:** M. Lazareva, L. Renemane, L. Rubene-Kesele, V. V. Vinogradova, S. Cīpare, L. Ķīse, K. Šulte, A. Bērziņa, E. Rancāns

**Affiliations:** 1 Department of Psychiatry and Narcology; 2 Department of Obstetrics and Gynaecology, Rīga Stradiņš University, Riga, Latvia

## Abstract

**Introduction:**

Postpartum depression has emerged as one of the most socially significant medical concerns, exerting a profound impact on maternal health, family relations, and child development (McNab et al. BMC Pregnancy and Childbirth 2022, 22(1):342; Slomian et al., Women’s Health 2019, 15:55). Exploring associations between the prevalence of depressive symptoms and various clinical and sociodemographic factors may provide valuable insights for the development of targeted screening strategies, thereby ensuring appropriate care and support for women in fulfilling their role as mothers.

**Objectives:**

Our study aimed to provide the first systematic assessment of postpartum depressive symptom prevalence, associated sociodemographic and clinical characteristics of women in outpatient care in Latvia.

**Methods:**

All women aged 18 years and older who consecutively attended the country’s largest Riga Maternity Hospital’s outpatient unit 4-6 weeks postpartum were screened with the Patient Health Questionnaire-9 (PHQ-9). Based on sensitivity and specificity analysis, a cut-off score of 8 was identified as the optimal threshold for the PHQ-9. Sociodemographic data were collected via self-administered questionnaires; clinical data were extracted from medical records. Group differences in PHQ-9 score distributions were analyzed using chi-square tests.

**Results:**

Over one year, 272 women aged 18-49 (mean 30.66 ± 5.59) were assessed. Depressive symptoms (PHQ-9 ≥8) were observed in 18.01%. Birth complications were significantly associated with higher PHQ-9 scores (p=0.031), and self-reported sleep problems showed a strong association (p=0.010). Mode of delivery (p=0.184), infant health problems (p=0.172), difficult infant temperament (p=0.124), and stressful events during pregnancy (p=0.100) showed non-significant trends. No significant differences were observed with respect to age, residence, ethnicity, education, marital and employment status, income, or other clinical factors (all p>0.05).

**Conclusions:**

Postpartum depressive symptoms were present in nearly one-fifth of women, underscoring the relevance of mental health in perinatal care. Birth complications and sleep problems emerged as significant associated factors, while other perinatal and psychosocial variables showed non-significant trends. These findings underscore the importance of systematic screening and early identification of women at risk, as well as the need for further studies to elucidate causal pathways and inform effective prevention and intervention strategies.

**Disclosure of Interest:**

None Declared

